# THE MEDICARE ANNUAL WELLNESS VISIT: POTENTIAL FOR EMBEDDED INTEGRATION OF THE PATIENT AND FAMILY PERSPECTIVE

**DOI:** 10.1093/geroni/igad104.0574

**Published:** 2023-12-21

**Authors:** Danielle Powell, M J Wu, Kelly Gleason, Stephanie Nothelle, Esther Oh, Nicholas Reed, Jennifer Wolff

**Affiliations:** University of Maryland, College Park, Maryland, United States; Johns Hopkins University, Baltimore, Maryland, United States; Johns Hopkins, Baltimore, Maryland, United States; Johns Hopkins School of Medicine, Baltimore, Maryland, United States; Division of Geriatric Medicine, Johns Hopkins University School of Medicine, Baltimore, Maryland, United States; Johns Hopkins Bloomberg School of Public Health, Baltimore, Maryland, United States; Johns Hopkins University, Baltimore, Maryland, United States

## Abstract

Patient portals are secure online platforms that provide an opportunity for patients to actively participate in their care by viewing their health information, messaging with clinicians, and completing health risk assessments, like the Medicare Annual Wellness visit (AWV). Using electronic medical record and patient portal (MyChart) data (October 3, 2021-October 2, 2022) from a large integrated academic medical center, we examine uptake of the AWV among Medicare beneficiaries 65+ years with 2 or more primary care visits. We then examine whether responses to the AWV health risk assessment differ by whether patients are registered for and actively use the patient portal. Among 11,378 eligible beneficiaries, 7,194 had an AWV billed (62% female, 69% White, 8.7% dementia, 78% patient portal users). Among those billed, 98% answered at least 1 health risk assessment question +/- 30 days of billing, and 76% completed questions related to all risk assessment themes. Nearly 8 in 10 (79.6%) older adults who completed the AWV were patient portal users. While highly active overall, completion of the AWV and health risk assessment was higher for active patient portal users. Compared to non-active portal users, active users were less likely to report difficulty with ADLs, transportation, chores, mobility issues, vision concerns, living alone, and seeing a dentist. Evaluation of billed AWV alone does not fully characterize completion and care provided for the preventive care visit. Systematic incorporation of the patient portal may aid in integration and dissemination of preventive care.

